# Supplementary material for: Drug Resistance Predictions Based on a Directed Flag Transformer
Source: Adv Sci (Weinh). 2025 Jul 3;12(36):e02756. doi: 10.1002/advs.202502756 (PMC12463116; doi:10.1002/advs.202502756)
Supplement: Supplementary file 1 — Supporting Information [file ADVS-12-e02756-s001.pdf]

ADVANCED SCIENCE
Open Access

Supporting Information

for *Adv. Sci.*, DOI 10.1002/advs.202502756

Drug Resistance Predictions Based on a Directed Flag Transformer

Dong Chen, Gengzhuo Liu, Hongyan Du, Benjamin Jones, Junjie Wee, Rui Wang, Jiahui Chen, Jana Shen and Guo-Wei Wei**

Supporting Information: Drug Resistance Predictions Based on a Directed Flag Transformer

Dong Chen,[†] Gengzhuo Liu,[†] Hongyan Du,[†] Benjamin Jones,[†] Junjie Wee,[†] Rui Wang,[‡] Jiahui Chen,[¶] Jana Shen,[*,§] and Guo-Wei Wei[*,‖]

† *Department of Mathematics, Michigan State University, MI, 48824, USA*

‡ *Simons Center for Computational Physical Chemistry, New York University, New York, NY, 10003*

¶ *Department of Mathematical Sciences, University of Arkansas, Fayetteville, AR 72701*

§ *Department of Pharmaceutical Sciences, University of Maryland School of Pharmacy, Baltimore, MD 21201*

‖ *Department of Mathematics & Department of Electrical and Computer Engineering & Department of Biochemistry and Molecular Biology, Michigan State University, MI, 48824, USA*

E-mail: Jana.Shen@rx.umaryland.edu; weig@msu.edu

Table of Contents

- Supplementary Tables
- Supplementary Figures
- Supplementary Mathematical Methods
- Supplementary Computational Methods and Protocols
- Supplementary Notes

Supplementary Tables

Table S1: Experimental data of SARS-CoV/CoV-2 Mpro-ligand complexes (part I)

PDB	Proteins	Ligand	IC50(nM)	Ki(nM)	pK_i	Ref.
7SI9	SARS-CoV-2 Mpro	4WI	26.03	1.88	8.726	S1
8D4K	SARS-CoV-2 Mpro H172Y	K36	449.2	432.5	6.364	S1
8D4M	SARS-CoV-2 Mpro S144A	K36	139.8	65.01	7.187	S1
8DD1	SARS-CoV-2 Mpro H164N	K36	126.3	37.95	7.421	S1
8DD9	SARS-CoV-2 Mpro S144L	K36	1812	1847	5.734	S1
8DGB	SARS-CoV-2 Mpro Q192T	K36	237.2	119.7	6.922	S1
6WTT	SARS-CoV-2 Mpro	B1S	40.25	15.5	7.810	S1
6XHM	SARS-CoV-2 Mpro	V2M		0.27	9.569	S2
6XHL	SARS-CoV Mpro	V2M		4	8.398	S2
6XHN	SARS-CoV Mpro	V3D	15	5.38	8.269	S2
2ZU4	SARS-CoV Mpro	ZU3		38	7.420	S3
2ZU5	SARS-CoV Mpro	ZU5		400	6.398	S3
6M0K	SARS-CoV-2 Mpro	FJC	40		7.398	S4
6LZE	SARS-CoV-2 Mpro	FHR	53		7.276	S4
2Z94	SARS-CoV Mpro	TLD		1400	5.854	S5
2Z9K	SARS-CoV Mpro	DOZ		320	6.495	S5
2GX4	SARS-CoV Mpro	NOL		53	7.276	S6
5N5O	SARS-CoV Mpro	8O5	240		6.620	S7
2GZ7	SARS-CoV Mpro	D3F	300		6.523	S8
2GZ8	SARS-CoV Mpro	F3F	3000		5.523	S8
6WTK	SARS-CoV-2 Mpro	UED	400	171.4	6.766	S9
6WTJ	SARS-CoV-2 Mpro	K36	190	81.42	7.089	S9
3D62	SARS-CoV Mpro	959		400	6.398	S10
6XMK	SARS-CoV-2 Mpro	QYS	450		6.347	S11
6W2A	SARS-CoV Mpro	QYS	1100		5.959	S11
6XFN	SARS-CoV-2 Mpro	GOL	450		6.347	S12
6LNY	SARS-CoV Mpro	EOC	508	90.5	7.043	S13
6Y2G	SARS-CoV-2 Mpro	O6K	670	900	6.046	S14
6M2N	SARS-CoV-2 Mpro	3WL	940		6.027	S15
6XA4	SARS-CoV-2 Mpro	GOL	0.41	0.6	9.222	S12
2V6N	SARS-CoV Mpro	MES		1380	5.860	S16
2Z94	SARS-CoV Mpro	TLD		1400	5.854	S5
5N19	SARS-CoV Mpro	D03	1950		5.710	S7
2OP9	SARS-CoV Mpro	WR1		2200	5.658	S17
3SN8	SARS-CoV Mpro	S89		8270	5.082	S18
2GZ8	SARS-CoV Mpro	F3F	3000		5.523	S8
4MDS	SARS-CoV Mpro	23H	6200		5.208	S19
2AMD	SARS-CoV Mpro	91N		6700	5.174	S20
3SNE	SARS-CoV Mpro	MES		2240	5.650	S18
1WOF	SARS-CoV Mpro	I12		10700	4.971	S20
3VB6	SARS-CoV Mpro	EDO	39000		4.409	S21
3SNC	SARS-CoV Mpro	DMS		72730	4.138	S18
3VB4	SARS-CoV Mpro	GOL	49000		4.310	S21
3VB7	SARS-CoV Mpro	GOL	49000		4.310	S21
4TWW	SARS-CoV Mpro	3A7	63000		4.201	S22
2ALV	SARS-CoV Mpro	CY6	70000		4.155	S23
2QIQ	SARS-CoV Mpro	CYV	80000		4.097	S24
4TWY	SARS-CoV Mpro	3BL	108000		3.967	S22

pK_i is calculated as $log_{10}(1/Ki)$ or $log_{10}(1/IC50)$ when K_i data are unavailable.

Table S2: Experimental data on SARS-CoV/CoV-2 Mpro-ligand complexes (part II) taken from Ref. Nguyen et al.[S25]

Complexes	logKa	Complexes	logKa	Complexes	pK_i
6y2g	6.174	narlaprevir	5.245	review-com12_6c	6.301
6m2n	6.027	CHEMBL50	4.621	CHEMBL1929017	5.245
7brp	5.380	CHEMBL2311733	4.350	CHEMBL3402617	4.166
6wtk	6.400	review-com8-2s	5.538	review-com11_5b	5.770
6wtj	6.720	CHEMBL2442075	4.650	CHEMBL380403	4.922
6m0k	7.420	CHEMBL538957	4.306	CHEMBL2402683	6.073
6lze	7.280	CHEMBL2402681	5.809	CHEMBL208908	4.445
6lo0	6.080	review-com11_5p	6.921	CHEMBL551569	3.939
6lny	6.290	CHEMBL2402499	5.230	review-com46_45	3.757
5n19	5.710	review3-com13-7	3.699	6y2g_13a	5.619
2gx4	7.280	CHEMBL213543	6.184	review-com12_6b	6.155
2amd	5.710	CHEMBL2311730	5.003	review-com13_11u	5.896
1wof	4.910	6lnq-12b	6.734	CHEMBL557699	3.800
6wtt	7.520	review-com13_11t	5.842	review-com8-2t	5.824
CHEMBL2402500	4.636	CHEMBL2402502	5.795	review3-com145_17	4.551
CHEMBL4130313	5.171	review-com8-2i	8.387	review-com8-2r	5.469
CHEMBL2402676	5.135	CHEMBL397154	5.003	CHEMBL3402615	3.873
CHEMBL2311700	4.408	review-com11_5o	6.097	CHEMBL2402678	6.154
CHEMBL2311729	4.122	review-com11_5h	8.201	CHEMBL2402677	6.162
review-com8-2m	6.125	CHEMBL2442077	5.164	review-com11_5j	7.523
review-com9_10-25f	4.538	review-com11_5l	5.174	CHEMBL2402501	6.338
CHEMBL235873	4.827	review3-com55-3	3.073	CHEMBL3402616	3.873
CHEMBL1230135	5.047	review-com11_5n	7.658	CHEMBL212080	7.240
CHEMBL2402680	6.250	review-com13_11s	6.620	CHEMBL2442082	5.208
CHEMBL378188	4.093	review-com8-2k	7.854	CHEMBL551181	3.235
CHEMBL555220	4.467	review-com8-2o	6.187	review-com11_5k	7.585
PSI	4.981	review3-com5	4.886	CHEMBL4299306	6.748
review-com54_30	4.620	CHEMBL2316337	5.340	CHEMBL2442076	5.384
CHEMBL539208	3.440	CHEMBL2311726	4.328	CHEMBL1929018	6.411
review-com13_11n	6.481	review-com13_11q	5.203	review-com8-2u	5.125
CHEMBL477164	6.697	CHEMBL2402684	5.494	CHEMBL1405735	5.655
review-com11_5e	6.796	review3-com61_5b	4.699	CHEMBL1929021	5.003
CHEMBL151	4.702	CHEMBL406516	5.003	review-com13_11p	4.971
CHEMBL2311705	4.298	review-com9_10-26k	6.076	CHEMBL2311709	5.223
CHEMBL2402507	5.494	CHEMBL553172	3.873	6lnq-12a	6.162
PMC2597651_4	6.294	PMC2597651_3	6.418	CHEMBL2401746	6.213
review-com13_11o	5.071	review-com8-2p	8.523	CHEMBL549695	6.220
6y2g_11r	6.748	CHEMBL2442079	5.113	review3-com4	4.409
CHEMBL2402508	6.374	CHEMBL3402619	4.247	review-com8-2n	7.959
CHEMBL2442074	5.039	CalpainXII	6.345	CHEMBL537916	3.528
review-com8-2l	5.721	review-com11_5c	7.187	review-com11_5r	6.167
review-com11_5g	6.481	CHEMBL2311731	5.259	CHEMBL1929022	6.470
CHEMBL2402682	5.076	CHEMBL12798	3.682	review3-com145_16r	5.824
review-com11_5a	5.569	calpeptin	4.973	CHEMBL63354	4.856
review-com8-2j	7.658	CHEMBL1929015	4.210	MG-132	5.406
CHEMBL2402685	6.411	CHEMBL28	3.550	review-com11_5d	7.174
review3-com13-3	4.097	review3-com61_5a	4.824	CHEMBL2402686	6.484
CHEMBL377324	4.518	CHEMBL2316336	4.408	CHEMBL1929020	6.572
review-com11_5i	7.319	CHEMBL2442073	5.421	review-com54_2	6.222
CHEMBL2402503	5.765	CHEMBL2311727	5.281	CHEMBL555061	4.680
CHEMBL2402504	4.540	CHEMBL2402692	6.184	CHEMBL277716	5.648
review-com54_33	4.620	MG-115	5.501		

Table S3: Five-fold cross-validation performance of the DFFormer-seq model on the SARS-CoV-2/CoV Mpro dataset.

**Fold**	**1**	**2**	**3**	**4**	**5**	**Mean**^{a}	**Mean**^{b}
**PCC**	0.642	0.597	0.570	0.735	0.723	0.654	0.644
**RMSE**	1.382	1.381	1.326	1.189	1.042	1.264	1.273

The RMSEs are given in kcal/mol. Mean^a gives the average performance across five folds. Mean^b means the performance on all predictions combined.

Table S4: Predicted binding affinity (BA) changes (10 × kcal/mol) due to single-point mutations at 19 binding site residues as well as P132 (the Omicron variant mutation site)

Residue	A	R	N	D	C	Q	E	G	H	I
M49	-7.4e-1	-2.3e-2	-8.1e-1	-1.4	-1.2	-6.4e-1	-9.9e-1	-1.1e-1	-7.1e-2	-4.8e-1
Y54	1.2	-1.2	1.4	1.0	4.9e-1	8.4e-1	1.2	4.7e-1	1.6	1.6
F140	-7.1e-1	-1.4	-1.6	-3.1	-3.8e-1	-1.5	-2.8	-4.6e-1	-1.3	-1.3
L141	5.9e-1	-6.1e-1	2.1e-1	-7.5e-1	-3.2e-1	4.6e-1	3.5e-1	-4.2e-05	6.8e-1	2.5e-1
N142	1.1	-2.1	n/a	-1.3	-1.6	6.0e-1	9.4e-2	-2.4	8.2e-1	3.9e-1
G143	-1.0	-1.2	-2.0	-2.4	-4.2e-1	-2.4e-1	-1.4	n/a	-8.5e-1	-3.8e-1
S144	7.8e-1	-2.0	-1.5	-1.8	-3.4	-2.5	-3.7	-4.2e-1	-1.2	-1.4
H163	1.8e-1	7.2e-1	-5.7e-1	-1.4	-1.9	-7.8e-1	-1.5	-9.3e-1	n/a	-1.2e-1
H164	5.6e-1	1.7e-1	1.6	-2.1e-1	-3.7e-1	-5.7e-1	-2.0e-1	-1.7	n/a	8.3e-1
M165	-2.7	-2.9	-3.3	-2.2	-2.0	-2.2	-2.5	-4.7	-1.4	2.8
E166	-2.6e-2	7.3e-1	6.4e-1	-1.3	3.7e-1	-1.5e-1	n/a	6.4e-1	1.9	2.2
L167	1.8e-2	-2.0	-2.3	-1.4	-7.1e-1	-2.8	-1.6	-9.1e-1	-1.2	-4.0e-1
P168	8.1e-1	1.9e-1	4.2e-1	4.2e-1	2.4	2.7e-1	6.7e-1	1.0	6.0e-1	1.3
H172	-3.7e-1	6.1e-1	2.3e-1	-1.6	-1.1	1.6e-1	-3.8e-1	-1.3	n/a	1.8
D187	-3.3e-1	-8.0e-1	8.1e-1	n/a	-4.3e-2	1.2	-3.5e-1	3.6e-1	9.4e-1	1.3
R188	-4.4e-1	n/a	6.6e-1	1.4e-1	-2.8	-1.6e-1	5.1e-2	-8.2e-2	9.3e-1	6.8e-1
Q189	-1.2	-7.0e-2	2.8e-1	1.1	-7.5e-1	n/a	-2.4e-1	-2.5	1.0	3.4e-1
T190	-1.6	8.6e-1	1.2	9.8e-1	1.9	4.9e-1	3.6e-1	-9.9e-1	2.5	1.0
Q192	5.8e-1	8.3e-1	2.1e-1	1.1	3.4	n/a	5.4e-1	-1.6e-1	1.6	1.6
**P132**	6.4e-1	4.2e-1	1.1	4.4e-1	-2.4e-1	1.3	3.9e-1	-6.5e-1	1.8	-4.9e-1

Residue	L	K	M	F	P	S	T	W	Y	V
M49	-1.6	-3.2e-1	n/a	6.7e-1	-1.6	-2.9e-1	-1.6	3.4e-1	5.7e-1	-5.5e-1
Y54	-1.5e-1	1.2	2.2	1.9	6.2e-1	1.2	5.6e-1	1.7	n/a	1.7
F140	-1.4	-4.8e-1	-4.8e-1	n/a	-2.6	-1.0	-6.4e-1	1.9e-1	-1.1	2.4e-1
L141	n/a	-4.3e-2	2.0	6.8e-1	-1.4	1.4e-1	-7.2e-1	4.3e-1	-5.6e-1	6.4e-2
N142	-2.4e-1	-8.2e-1	1.6	-3.7e-2	-1.1	4.1e-1	-1.5	-1.7	1.4e-1	5.1e-1
G143	-7.4e-1	-6.8e-1	3.9e-1	-1.0	-1.0	-1.3	-1.5	-4.4e-1	-5.0e-2	-7.1e-1
S144	-5.3	-1.7	-3.1	-3.0	-4.4	n/a	-9.6e-1	-1.3	-2.0	-1.4
H163	-1.7e-2	7.4e-1	-8.1e-2	5.4e-1	-1.1	-8.9e-1	-4.7e-1	-1.4e-1	1.1	-3.8e-1
H164	6.0e-2	5.5e-1	-3.7e-1	8.2e-1	-6.5e-1	1.5e-2	-4.3e-1	4.5e-1	-1.3	8.2e-1
M165	2.7	-2.8	n/a	4.9	-4.0	-3.4	-2.0	-9.5e-1	-4.2	7.4e-1
E166	1.4	1.7	1.5	4.3	1.5	-2.5e-1	2.9	4.3	3.4	2.2
L167	n/a	-2.6	1.7	1.1	-1.2	-1.0	-1.1	2.4	-2.7e-1	-9.0e-2
P168	7.4e-1	1.2	2.3	1.3	n/a	1.2	1.1	1.8e-1	5.6e-1	1.5
H172	-1.8e-1	5.2e-1	1.7	-1.3e-1	-2.0	-1.9	-1.4	4.1e-1	-4.3	1.3
D187	8.6e-1	6.2e-1	1.6	7.6e-1	-2.3	-8.4e-1	-1.6	2.9	1.7	5.0e-1
R188	-6.8e-1	5.2e-1	1.9	-2.0e-1	-2.6	-3.1	-2.1e-1	-1.4e-1	-7.9e-1	4.1e-1
Q189	-7.9e-1	-3.2e-1	7.4e-1	8.8e-1	-8.7e-1	2.6e-1	-2.1e-1	4.9e-1	3.6e-1	6.9e-2
T190	9.3e-1	6.4e-1	2.0	2.2	4.1e-1	1.6	n/a	2.6e-1	1.6	8.2e-1
Q192	9.0e-1	-1.6	4.5	1.1	7.7e-1	6.7e-1	-1.6	1.4	9.0e-1	3.2
**P132**	-1.1e-1	1.2	3.0e-1	9.0e-1	n/a	5.5e-1	1.2	1.7	2.2e-1	-6.0e-1

Each residue at the nirmatrelvir binding site or the Omicron variant mutation site (P132, in bold font) is evaluated for 19 potential mutations, whereby the BA change (10 × kcal/mol) relative to the WT is calculated as ΔBA=BA(mut)-BA(WT). Note, BA is related to pK_i as 1.363*pK_i. The entries for the WT are shown as n/a.

Table S5: Experimental IC50 and K_i values of nirmatrelvir against mutants of SARS-CoV-2 Mpro and labels for potential drug resistance

Mutation	IC50(nM)	pIC50	K_i(nM)	pK_i	Label	Mutation	IC50(nM)	pIC50	K_i(nM)	pK_i	Label
WT	26.03	7.585	1.88	8.726	n/a	H172L	3380	5.471	3135	5.504	1
N142Y	14.9	7.827			0	H172M	699	6.156	290.6	6.537	1
N142T	18.73	7.727			0	H172A	374.7	6.426	213.8	6.670	1
N142K	13.93	7.856			0	H172I	10000	5.000	10000	5.000	1
N142D	24.3	7.614			0	H172N	1827	5.738	1613	5.792	1
N142L	15.29	7.816			0	H172S	5650	5.248	2204	5.657	1
N142S	25.97	7.586			0	H172Q	152.1	6.818	70.54	7.152	1
N142I	17.11	7.767			0	H172K	1030	5.987			1
N142H	19.65	7.707			0	H172R	10000	5.000			1
N142M	19.01	7.721			0	H172G	810.2	6.091			1
S144D	8532	5.069			1	H172C	3056	5.515			1
S144K	10000	5.000	10000	5.000	1	H172V	10000	5.000			1
S144R	6134	5.212	2190	5.660	1	H172F	212.2	6.673	46.6	7.332	1
S144M	175	6.757	79.26	7.101	1	H172T	10000	5.000	12672	4.897	1
S144F	133.3	6.875	47.23	7.326	1	H172Y	279.3	6.554	275.1	6.561	1
S144G	96.55	7.015	27.98	7.553	1	H172D	266.1	6.575	170.6	6.768	1
S144A	171.1	6.767	36.43	7.439	1	H172E	10000	5.000			1
S144Y	61.49	7.211	34.09	7.467	1 (0)	Q189F	34.02	7.468			0
S144L	5364	5.271	3952	5.403	1	Q189R	37.82	7.422			0
S144T	353.7	6.451	207.1	6.684	1	Q189H	32.71	7.485			0
S144P	10000	5.000	10000	5.000	1	Q189L	39.25	7.406			0
S144W	289.6	6.538	143.8	6.842	1	Q189P	14.08	7.851			0
S144E	423.4	6.373	153.2	6.815	1	Q189S	25.34	7.596			0
S144V	1442	5.841	858.9	6.066	1	Q189E	49.71	7.304	4.51	8.346	0
S144H	650.5	6.187	402.2	6.396	1	Q189K	39.31	7.405	14.6	7.836	0
S144Q	831.2	6.080	576.8	6.239	1	Q192F	92.68	7.033	85.5	7.068	1
H164N	32.85	7.483	3.58	8.446	0	Q192K	219.1	6.659	88.9	7.051	1
M165T	94.68	7.024	52.68	7.278	1	Q192L	121.9	6.914	70.99	7.149	1
M165Y	10462	4.980	7216	5.142	1	Q192S	217.3	6.663	75.56	7.122	1
M165F	1336	5.874			1	Q192A	140.7	6.852	58.1	7.236	1
M165H	10000	5.000			1	Q192I	109.9	6.959	43.53	7.361	1
M165P	10000	5.000			1	Q192P	135.1	6.869	76.19	7.118	1
M165R	10000	5.000			1	Q192G	319.1	6.496	106.3	6.973	1
M165W	10000	5.000			1	Q192E	193.1	6.714	114.4	6.942	1
M165K	10000	5.000			1	Q192H	169.4	6.771	80.69	7.093	1
M165L	22.62	7.646	1.04	8.983	0	Q192T	102.6	6.989	45.69	7.340	1
M165I	27.38	7.563	2.02	8.695	0	Q192V	95.58	7.020	30.87	7.510	1
M165V	23.96	7.621			0	Q192W	65.94	7.181	43.65	7.360	1
M165A	23.28	7.633			0	Q192C	96.82	7.014	54.12	7.267	1
M165G	243.5	6.614			0	Q192D	271.5	6.566			1
M165C	24.84	7.605			0	Q192Y	10000	5.000			1
M165D	147.6	6.831			0	Q192N	73.69	7.133			0
E166I	10000	5.000			1	Q192R	229.6	6.639			0
E166G	92.15	7.036	30.89	7.510	1	M49L	18.5	7.733	1.57	8.804	0
E166V	10000	5.000	10384	4.984	1	M49T	15.76	7.802	2	8.699	0
E166A	152	6.818	89.29	7.049	1	M49V	23.48	7.629			0
E166Y	3711	5.431			1	M49I	26.54	7.576			0
E166H	9598	5.018			1						
E166K	1994	5.700			1						
E166Q	27.87	7.555	8.37	8.077	0						
E166L	214.2	6.669			0						

Experimental data taken from Hu et al.,[S1] with double mutants excluded. pK_i =-$\log_{10} K_i$ and pIC50 = -$\log_{10}$IC50. A mutation is labeled as potentially drug resistance if pK_i(mut)−pK_i(WT) $<$ -1 or pIC50(mut)−pIC50(WT) $<$ -1 (if K_i is unavailable).

Table S6: Potential drug-resistance mutations predicted by CAPTURE

Mutation	ΔBA	Status	Mutation	ΔBA	Status
M49L	-0.16	FP	H164Y	-0.13	TBT
M49T	-0.16	FP	L167S	-0.10	TBT
**F140L**	-0.14	TP	**H172Y**	-0.43	TP
N142D	-0.13	FP	R188S	-0.31	TBT
N142T	-0.15	FP	T190A	-0.16	TBT
G143S	-0.13	TBT			

Predicted ΔBAs are given in kcal/mol. Mutations confirmed by experiments[S1,S26,S27] are highlighted. The status column lists three values: true positive, false positive, and to be tested (TBT) for predictions awaiting experimental verification.

Table S7: Performance of DFFormer-seq on predicting nirmatrelvir-resistance mutations in Mpro without and with second-stage fine-tuning.

**Model Version**	**AUROC**	**Recall**	**Precision**	**Accuracy**
First-stage fine-tuned	0.47	0.27	0.75	0.63
Second-stage fine-tuned	0.60	0.57	0.71	0.77

Table S8: Representative mutations within 15 Å of nirmatrelvir showing increased frequency during Period #5 (Oct-Dec 2022).

Mutation Site	Protein Site	Total Frequency	Change	Mutation Site	Protein Site	Total Frequency	Change
10055:A→G	S1G	382	1	10588:A→C	E178D	28	1
10065:G→A	R4K	416	5	10588:A→T	E178D	9	2
10089:A→G	K12R	231	1	10592:A→G	N180D	98	1
10091:G→A	V13I	125	2	10592:A→T	N180Y	6	1
10092:T→C	V13A	20	1	10604:C→T	P184S	1249	12
10097:G→A	G15S	10864	11	10605:C→A	P184H	1616	2
10097:G→T	G15C	949	3	10605:C→T	P184L	983	6
10103:A→G	M17V	943	3	10610:G→T	V186F	990	5
10104:T→C	M17T	78	1	10623:C→A	T190K	5	1
10105:G→A	M17I	145	3	10623:C→T	T190I	915	2
10105:G→T	M17I	1217	13	10625:G→T	A191S	62	3
10106:G→A	V18I	377	1	10626:C→T	A191V	4021	32
10113:T→C	V20A	85	5	10631:G→A	A193T	724	3
10116:C→T	T21I	3445	19	10631:G→T	A193S	101	8
10125:C→T	T24I	980	19	10632:C→T	A193V	1245	5
10128:C→T	T24I	303	303	10634:G→T	A193S	167	167
10128:C→T	T25I	303	5	10634:G→T	A194S	167	3
10142:C→A	L30I	54	13	10640:A→G	T196A	94	1
10151:G→A	D33N	21	1	10641:C→T	T196M	3836	2
10157:G→A	V35I	534	4	10646:A→G	T198A	757	5
10157:G→T	V35L	83	1	10646:A→T	T198S	15	2
10160:G→A	V35I	132	132	10647:C→T	T198I	444	4
10201:G→T	M49I	944	1	10649:A→G	T198A	231	2
10202:C→T	L50F	1918	16	10649:A→G	T199A	231	2
10206:A→G	N51S	83	1	10650:C→T	T198I	876	876
10418:C→T	P122S	101	2	10650:C→T	T199I	876	2
10440:C→T	A129V	4063	22	10664:G→A	V204I	65	1
10448:C→T	P132S	3903	6	10667:T→G	L205V	4922	162
10449:C→A	P132H	206412	11514	10868:C→A	L271M	73	1
10449:C→T	P132L	4929	37	10868:C→A	L272M	73	1
10458:C→T	T135I	721	1	10871:C→A	Q273K	347	3
10496:G→A	V148I	112	4	10872:A→T	Q273L	244	1
10512:A→G	D153G	170	2	10874:A→G	N274D	935	2
10515:A→G	Y154C	5	1	10875:A→G	N274S	1415	5
10517:G→A	D155N	83	1	10901:G→A	G283S	570	5
10519:C→A	D155E	28	1	10902:G→A	G283D	164	1
10521:G→A	C156Y	455	7	10904:A→G	S284G	608	1
10523:G→A	V157I	151	2	10905:G→A	S284N	42	1
10523:G→C	V157L	5758	12	10908:C→T	A285V	963	3
10526:T→A	S158T	91	10	10928:A→G	T292A	31	1
10529:T→C	F159L	251	1	10943:G→A	V296I	1432	7
10540:G→T	M162I	1005	3	10943:G→A	V297I	1432	7
10560:C→G	T169S	201	1	10947:G→A	R298K	178	1

The full list is available at `https://github.com/WeilabMSU/CAPTURE` and the latest frequency data is posted at `https://users.math.msu.edu/users/weig/SARS-CoV-2_Mutation_Tracker.html`.

Supplementary Figures

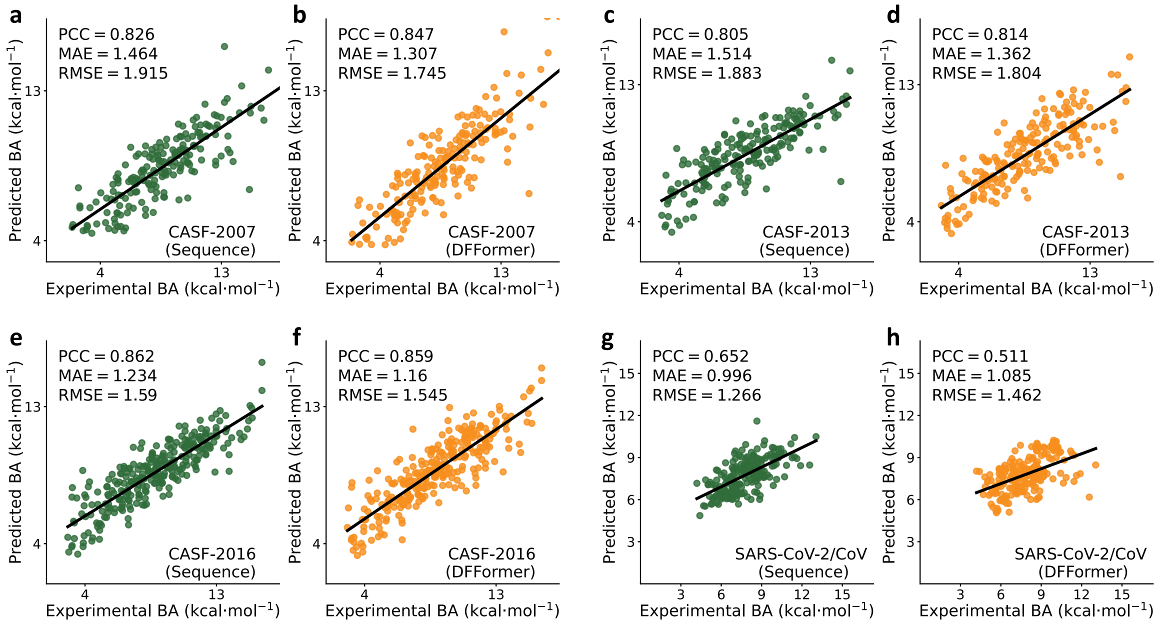

Figure S1: Performances of DFFormer and Seq-trees in predicting protein-ligand BAs from the CASF and SARS-CoV/CoV-2 Mpro datasets. Comparison between the predicted and experimental BAs using the Seq-ML model (**a, c, e, g**) and the DFFormer model (**b, d, f, h**). Note, for the SARS-CoV/CoV-2 Mpro dataset, the 5-fold CV is shown.

Supplementary Mathematical Methods

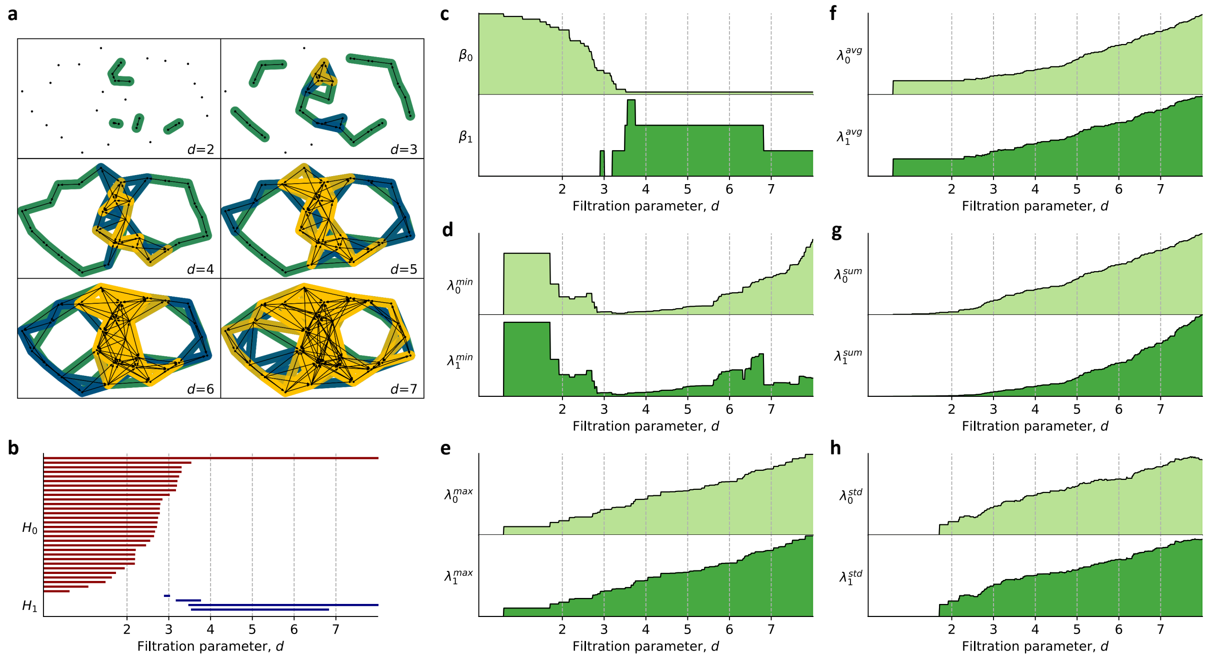

Figure S2: Analysis of persistent directed flag Laplacian versus persistent directed flag homology for the point cloud shown in main text Figure 5**a**. **a**, How varying the filtration parameter alters the connectivity of the point cloud, leading to a sequence of directed flag complexes that represent different topological structures. **b**, The barcodes for persistent directed flag homology groups H_0 and H_1 at the 0th and 1st dimensions, respectively, highlighting their changes due to the filtration (or scale) parameter d. **c**, Visualization of the variations in the topological invariants at the 0th (β_0) and 1st (β_1) dimensions of the persistent directed flag complex as a function of d. **d**, Representation of the nonzero minimum non-harmonic spectra of the persistent directed flag Laplacian at the 0th and 1st dimensions (λ_0^{min} and λ_1^{min}), emphasizing their dependence on d. **e**, Representation of the maximum non-harmonic spectra of the persistent directed flag Laplacian at the 0th and 1st dimensions (λ_0^{max} and λ_1^{max}), emphasizing their dependence on d. **f**, The average values of nonzero spectra for the persistent directed flag Laplacian in both the 0th and 1st dimensions (λ_0^{avg} and λ_1^{avg}). **g**, The standard deviation of nonzero spectra for the persistent directed flag Laplacian in the 0th and 1st dimensions (λ_0^{std} and λ_1^{std}). **h**, The sum of nonzero spectra values for the persistent directed flag Laplacian in the 0th and 1st dimensions (λ_0^{sum} and λ_1^{sum}).

Fig. S2 illustrates the persistent characteristics obtained from the non-harmonic spectra of a persistent directed flag Laplacian in both the 0th and 1st dimensions. By incrementally modifying d, a series of hierarchical data representations can be obtained (Fig. S2**a**). Panel **e** shows the progression of zero eigenvalue multiplicities in the corresponding Laplacian matrix as the filtration parameter (d) evolves, while Panel **f** and **g** show the changes in the nonzero minimum and maximum positive eigenvalue as d varies. Panel **d** shows the persistent barcodes for homology groups H_0 and H_1 in the 0th and 1st dimensions, respectively. The topological invariants,

specifically the Betti numbers, correspond to the frequency of the zero eigenvalue in the directed flag Laplacian for a specific d. It is important to note that these persistent attributes change with varying d, thereby capturing both topological and geometric data in a multiscale fashion.

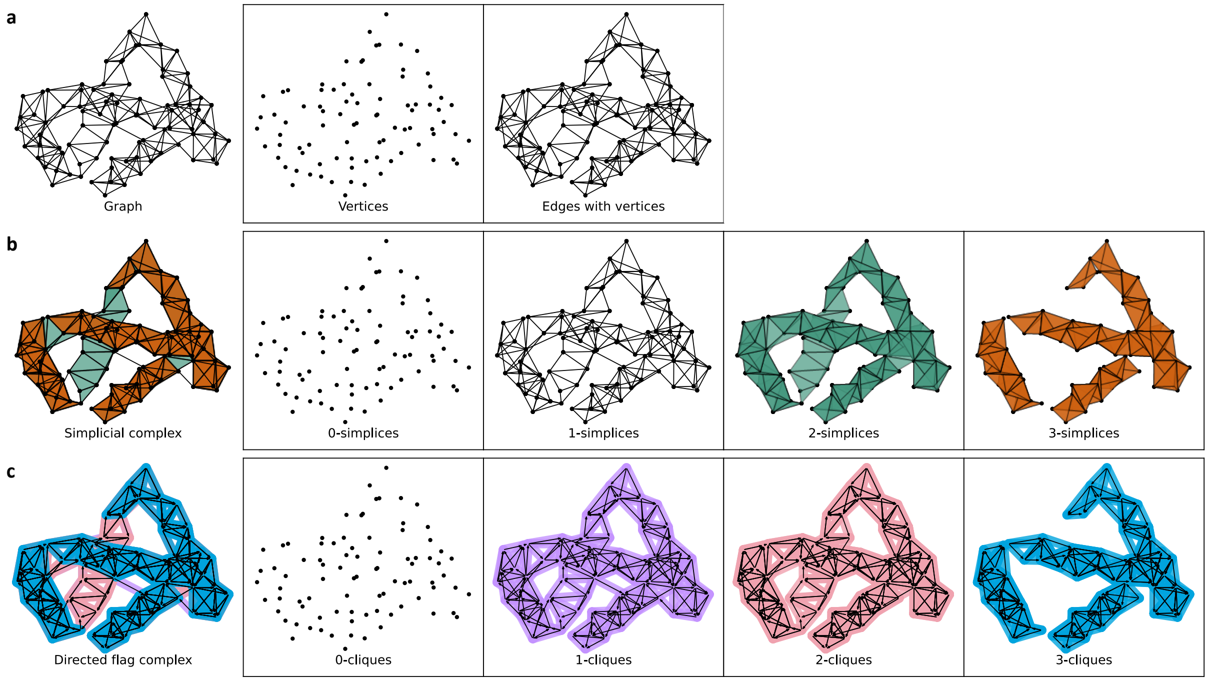

Figure S3: Illustration of different representations of a protein based on the C_α atoms (PDBID: IMYL). **a** A graph representation of the structure. **b** The simplicial complex representation of the structure, including a list of 0-simplices, 1-simplices, 2-simplices, and 3-simplices in the complex. **c** A directed flag complex representation of the structure, along with a list of 0, 1, 2, and 3-clique within the directed flag complex.

Figure S3 shows a variety of topological representations of a protein based on C_α atoms (PDBID: IMYL) and a pairwise cutoff distance of 5 Å. In Panel **a**, the traditional graph representation of the protein structure is displayed. Building on this graph representation, the 0-simplices in the simplicial complex are equivalent to the vertices in the graph, and the 1-simplices correspond to edges connecting these vertices, as demonstrated in the second and third columns of Panels **a** and **b**. Additionally, higher-dimensional simplices within the simplicial complex reveal more complex aspects of the structure. For example, 3-simplices can approximate the structure of alpha helices. With the directional attributes, directed flag complexes offer a more comprehensive representation than simplicial complexes. Directed flag complexes incorporate cliques of various dimensions, thus capturing multilevel structural information, as depicted in Panel **c**.

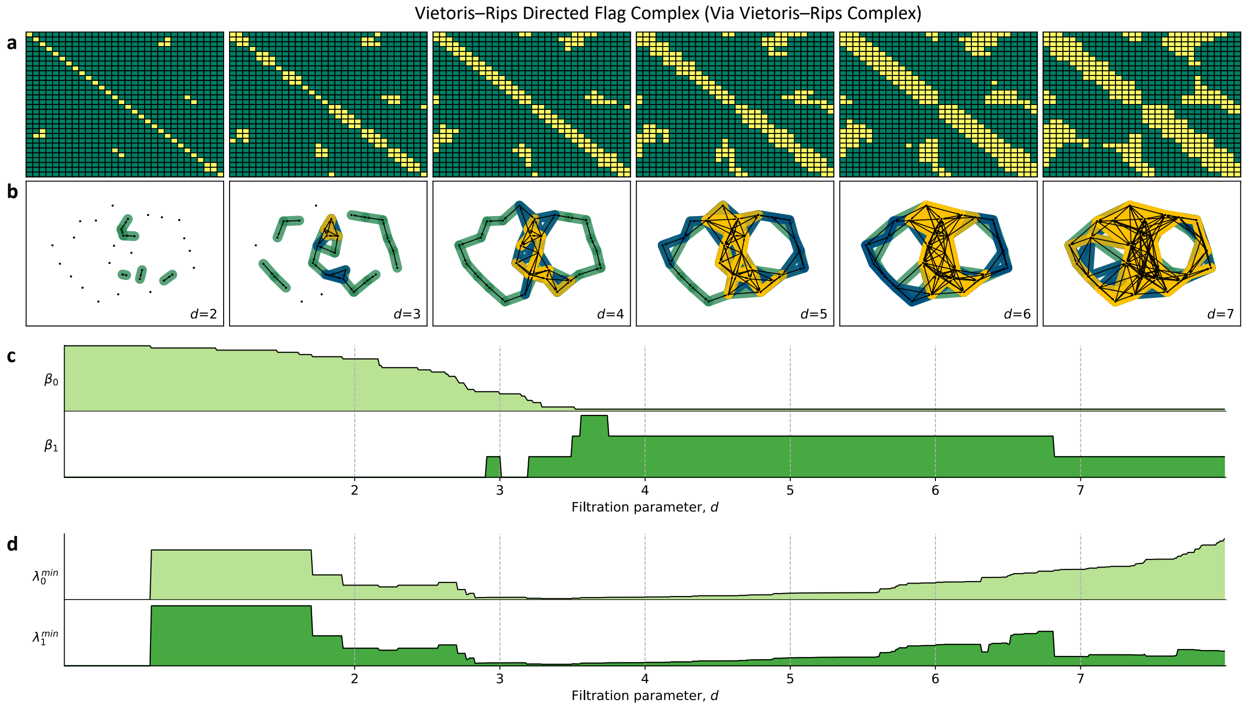

Figure S4: Illustration of Vietoris-Rips directed flag complex across various scales for the point cloud shown in the main text Figure 5**a**. **a** The adjacency matrices of the point cloud at different scales, are characterized by the filtration parameter d. Yellow entries represent connected points with a distance below the chosen cutoff, whereas green entries mark points that are not connected. **b** The Vietoris-Rips directed flag complexes constructed at multiple scales are displayed with d = 2, 3, 4, 5, 6, or 7. **c** The persistent Betti numbers, labeled as β_i for $i = 0$ and $i = 1$. The specific scales at which these Betti numbers occur are indicated by vertical dashed lines. **d** The minimum non-harmonic spectra (non-zero) of the persistent directed flag Laplacian in both the 0th and 1st dimensions (λ_0^{min} and λ_1^{min}), emphasizing how these values vary with the scale parameter d.

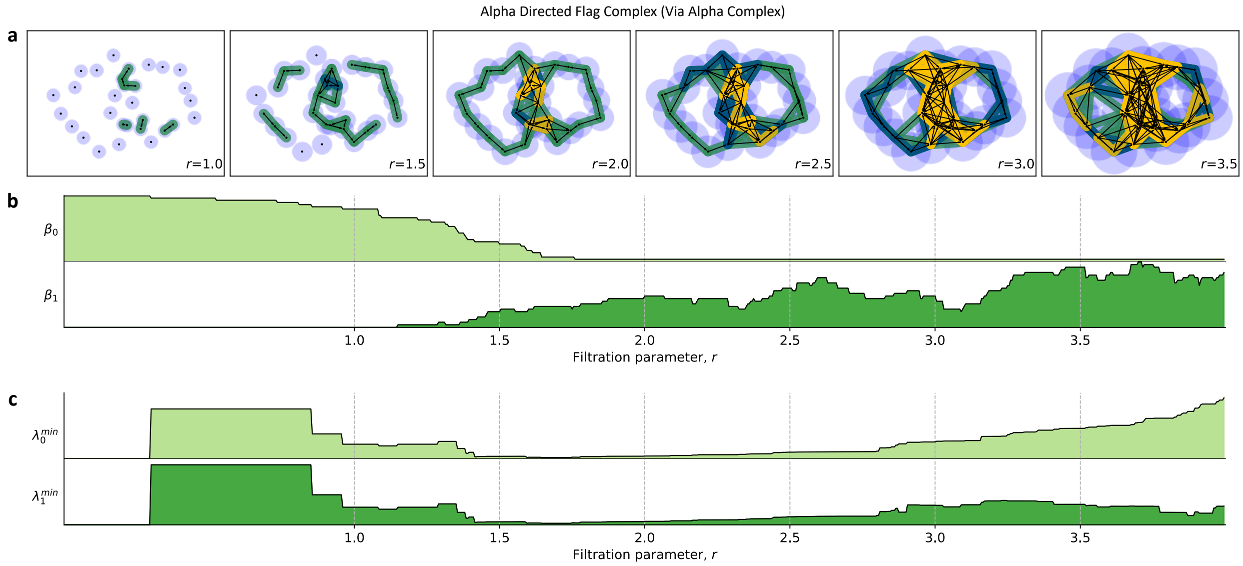

Figure S5: Illustration of building an alpha directed flag complex from the alpha complex with varying scale parameters for the point cloud shown in Figure 5**a**. **a** The constructed alpha directed flag complexes at a given filtration parameter, i.e., $r = 1.0$, $r = 1.5$, $d = 2.0$, $d = 2.5$, $d = 3.0$, and $d = 3.5$. **b** The persistent Betti numbers of alpha directed flag complexes, $\beta_i, i = 0, 1$. The vertical dash lines indicate the Betti numbers at a given filtration parameter. **c** Representation of nonzero minimum non-harmonic spectra of the persistent directed flag Laplacian at the 0th and 1st dimensions (λ_0^{min} and λ_1^{min}) for alpha directed flag complex, highlighting their dependence on the filtration parameter.

Directed flag topological sequence embedding

Directed flag complex. A directed flag complex is a conceptual extension of graphs, directed graphs, and simplicial complexes, serving as a combinatorial and topological structure derived from a directed graph (digraph). This framework generalizes the simplicial complex by incorporating directionality into the underlying graph. In Figure 5**b**, examples of the building blocks of the directed flag complex for various dimensions, such as 0-cliques, 1-cliques, 2-cliques, and 3-cliques, are presented. These structures embed directional information into the simplicial complex, contrasting with the 0-simplex (a single node), 1-simplex (a line segment), 2-simplex (a triangle), and 3-simplex (a tetrahedron) illustrated in Figure 5**c**.

In mathematical terms, given a directed graph $G = (V, E)$, where V is the set of vertices and $E \subseteq V \times V$ is the set of directed edges, the directed flag complex (or directed clique complex) of a directed graph G is a simplicial complex where the vertices of the directed flag complex correspond to the vertices of G, and a k-clique (also refer to directed k-simplex) in the directed flag complex corresponds to a directed $(k+1)$-clique in G. That is, a subset of $k+1$ vertices $\{v_0, v_1, \ldots, v_k\} \subset V$ such that $(v_i, v_j) \in E$ for all $i < j$. The clique here means the complete subgraph here.

Vietoris-Rips directed flag complex and alpha directed flag complex. The Vietoris-Rips (VR) complex and the alpha complex are highly regarded topological models for analyzing sets of data points. In cases where $\mathcal{K}$ is a VR complex or an alpha complex, the points that comprise a simplex in $\mathcal{K}$ inherently encapsulate geometric information, including both the size and orientation of the set of points. Drawing inspiration from the VR complex and the alpha complex, we introduce the Vietoris-Rips (VR) directed flag complex and the alpha directed flag complex, aimed at capturing this geometric information. To describe the geometry of simplices, a weight function $w : \mathcal{K} \to \mathbb{R}$ and a graded orientation function $\varrho_n : \mathcal{K}_n \to S_{n+1}$ for $n \geq 1$ are employed. In this context, S_n represents the permutation group with n elements. The VR/alpha

directed flag complex is formulated as follows:

$$\mathcal{DF}_\eta := \{S \times \varrho_*(S) \mid w(S) \leq \eta, S \in \mathcal{K}\}.$$

The VR/alpha directed flag complex can be viewed as an extension of the conventional VR/alpha complex. It reduces to the standard VR/alpha complex when the functions $w : \mathcal{K} \to \mathbb{R}$ and $\varrho_n : \mathcal{K}_n \to S_{n+1}$ are considered constant.

In this study, unless explicitly stated otherwise, all analyses are based on the Vietoris-Rips (VR) directed flag complex. Illustrative examples of the VR directed flag complex and the alpha directed flag complex can be seen in Figures S4 and S5.

Topological deep learning Topological deep learning (TDL) was introduced in 2017 for topology-based deep convolutional and multi-task neural networks for biomolecular property predictions.[S28] The early development was based on persistent homology,[S29,S30] a vital tool in topological data analysis.[S31,S32] TDL was the top winner in DR3 Grand Challenges, a worldwide annual competition series in computer-aided drug design,[S33,S34] demonstrating the power of algebraic topology in biomolecular modeling and prediction. Over the years, TDL has become an emerging paradigm in data science.[S35] However, persistent homology has many limitations.[S36] Some of these limitations were addressed by persistent Laplacians,[S37,S38] including persistent directed flag Laplacians.[S39]

Directed flag Laplacians and spectrum analysis. The combinatorial Laplacian is a key instrument in discrete geometry and algebraic topology, providing insights into the structure of various topological systems.[S36] These include simplicial complexes,[S37] directed flag complexes,[S39] cellular sheaves,[S40] path complexes,[S41,S42] hypergraphs,[S43] and hyperdigraphs.[S44] Analogous to how the graph Laplacian is utilized for examining graph properties (with graphs being equivalent to 1-simplices), the combinatorial Laplacian is employed for exploring attributes of simplicial and directed flag complexes. The eigenvalues of the graph Laplacian reveal connectivity aspects of

the graph. For instance, the second-smallest eigenvalue, known as the Fiedler vector, indicates the graph's algebraic connectivity. Similarly, the smallest positive eigenvalue, referred to as the spectral gap, has a significant relation to the Cheeger constant. The entirety of these eigenvalues forms the spectrum of the Laplacian operator.

The Laplacian matrix of a graph is defined as $\mathcal{L} = D - A$, where D is the degree matrix, and A is the adjacency matrix. In the context where the graph is considered as a one-dimensional (1D) simplicial complex, and taking B_1 as the matrix that represents the 1D boundary operator, we find that the graph Laplacian matrix can be accurately formulated as $\mathcal{L} = B_1 B_1^T$. This observation paves the way for extending the Laplacian operator to higher dimensions through the use of the boundary operator, culminating in the Laplacian operator for simplicial complexes. Suppose K represents a simplicial complex, and B_k signifies the matrix of its k-dimensional boundary operator. The Laplacian matrix in this context is given by:

$$\mathcal{L}_k = B_{k+1} B_{k+1}^T + B_k^T B_k. \tag{1}$$

In this equation, B_k^T is the transpose of B_k. The expression $B_k^T B_k$ reflects the connectivity that arises at the juncture of k-simplices at $(k-1)$-simplices. In contrast, $B_{k+1} B_{k+1}^T$ represents the interactions that occur due to the incorporation of k-simplices into $(k+1)$-simplices.

Recall that the topological characteristics of simplicial complexes or directed flag complexes are obtained from their chain complexes. The Laplacian operator can be defined from the perspective of chain complexes. Consider C_* as a chain complex with real coefficients, equipped with the differential $\partial_k : C_k \to C_{k-1}$. We assume that for each k, C_k is endowed with an inner product structure. As a result, the boundary operator ∂_k is paired with its adjoint operator ∂_k^*. The *combinatorial Laplacian* $\Delta_k : C_k \to C_k$ is then defined as

$$\Delta_k = \partial_{k+1} \circ \partial_{k+1}^* + \partial_k^* \circ \partial_k. \tag{2}$$

In particular, Δ_0 is given by $\partial_1 \circ \partial_1^*$. For each k, after selecting a standard orthonormal basis

for C_k, the corresponding representation matrix L_k of the Laplacian operator Δ_k, relative to this basis, is expressed as form in Equation 1, where B_k represents the matrix corresponding to the boundary operator ∂_k, as determined by multiplication to the left.[S45]

This combinatorial Laplacian expands upon the graph Laplacian, which primarily addresses properties of graphs, conceptualized as 1-simplicial complexes. In contrast, the combinatorial Laplacian extends the analysis to higher dimensions. Its eigenvalues and eigenvectors reveal significant geometric and topological details about the simplicial or directed flag complex. Given that the Laplacian matrix is positive semidefinite, all its eigenvalues are nonnegative. The zero eigenvalues, forming the harmonic spectrum, are particularly informative of the topological structure. Meanwhile, the non-zero eigenvalues, or the non-harmonic spectrum, convey the geometric aspects of the system. In the case of $\mathcal{L}_k$, the frequency of zero eigenvalues (indicating the number of occurrences of 0 as an eigenvalue) corresponds to the count of independent components and also to the topological invariants (β_k) in the k-dimensional space.[S46] For example, the zero multiplicity for $\mathcal{L}_0$ (i.e., β_0) represents the count of connected components in the graph (1-simplicial complex), for $\mathcal{L}_1$ (i.e., β_1) it denotes the number of cycles, and for $\mathcal{L}_2$, it signifies the number of cavities. The highest eigenvalue λ_k^{max} of $\mathcal{L}_k$ is bounded by the maximal count d_k of $k+1$-simplices sharing a common k-simplex (akin to the maximum degree of the graph for $\mathcal{L}_0$), so $0 \leq \lambda_k^{max} \leq 2d_k$. The smallest non-zero eigenvalue of $\mathcal{L}_k$, known as the spectral gap and denoted by λ_k^{min}, reflects the geometric structure of the system. This work utilizes various measures like the multiplicity of zero, the average, the standard deviation, the minimum, the maximum, and the sum of the positive eigenvalues of $\mathcal{L}_0$ for embedding given topological Laplacians.

Persistent directed flag Laplacians. Persistent Laplacians, also known as multiscale/persistent topological Laplacians, emerged from a collection of studies conducted on differential manifold settings[S47] and discrete point cloud scenarios.[S37] Central to the concept of persistent Laplacians,[S37,S44,S48] as well as in persistent homology,[S29,S30] is the process of filtration, which facilitates multiscale representation. This filtration, characterized by the scale parameter denoted as d,

is tailored according to the specific data structure under examination. For example, in the context of point cloud data (as shown in Figure 5**a**), this parameter typically represents the radius (or diameter) of a sphere. By incrementally modifying d, a series of hierarchical data representations can be obtained, as depicted in Figure S2**a**. Importantly, these representations extend beyond simplicial complexes to include structures such as directed flag complexes.[S39] For example, in the context of a distance matrix in which matrix elements denote distances between vertices, a filtration operation can be implemented. Here, a cutoff value is designated as the scale parameter, and if the distance between two vertices is less than this cutoff, an edge is established. As this cutoff value increases, a sequence of nested graphs is formed, where each graph, corresponding to a lower cutoff value, forms a subset of the graphs generated at higher cutoffs.

Similarly, nested simplicial complexes can be constructed using different complex formulations such as the Vietoris-Rips complex, the Čech complex, and the alpha complex. In this study, the focus is on the Vietoris-Rips complex. Mathematically, these nested simplicial complexes are represented as follows:

$$\emptyset \subseteq K_{d_0} \subseteq K_{d_1} \subseteq \cdots \subseteq K_{d_n} = K \tag{3}$$

Here, it holds that for any two values $d_i < d_j$, the complex K_{d_i} is a subset of K_{d_j}. This concept is also applicable to directed flag complexes, specifically through the Vietoris-Rips directed flag complex framework, where nested directed flag complexes are formed by defining directed edges in a particular manner.[S39] To illustrate the impact of varying the filtration parameters, Figure S2**a** demonstrates changes in point cloud connectivity from Figure 5**a**, resulting in a progression of directed flag complexes. The methodology for constructing the Vietoris-Rips directed flag complex is detailed in Figure S4. Furthermore, this work introduces the concept of the alpha directed flag complex, inspired by the alpha complex, as depicted in Figure S5.

During the progression of a filtration process, a series of chain complexes naturally emerges. Upon each filtration step, denoted d_i (with i serving as the step index), a distinct chain complex $C(K_{d_i}; G)$ is formed. In mathematical terms, a chain complex associated with a specific filtration step consists of a sequence of Abelian groups (or modules) connected by boundary

homomorphisms, which can be represented as follows:

$$\cdots \to C_{k+1}(K_{d_i};G) \xrightarrow{\partial_{k+1}^{d_i}} C_k(K_{d_i};G) \xrightarrow{\partial_k^{d_i}} C_{k-1}(K_{d_i};G) \to \cdots \quad (4)$$

In this sequence, $C_k(K_{d_i};G)$ denotes the chain group in the k-dimensional space at the specific filtration step d_i.

To provide a broader understanding, we will now present the Laplacian in a more comprehensive mathematical framework. Consider two real numbers, a and b, with $a \leq b$. Let chain complex C_*^a be a subset of C_*^b. The chain complexes in question could be derived from a variety of sources, such as filtration of simplicial complexes, directed flag complexes, or hyperdigraphs, to name a few. Furthermore, it is important to note that both C_*^a and C_*^b possess compatible inner product structures. Let $C_{k+1}^{a,b}$ be a set containing elements x in C_{k+1}^b such that the boundary operator ∂_{k+1}^b applied to x produces an element in C_k^a, formally expressed as $C_{k+1}^{a,b} = \{x \in C_{k+1}^b | \partial_{k+1}^b x \in C_k^a\}$.

The persistent boundary operator, denoted as $\partial_{k+1}^{a,b}$ and mapping from $C_{k+1}^{a,b}$ to C_k^a, is defined through the action $\partial_{k+1}^{a,b} x = \partial_{k+1}^b x$ for any x residing in $C_{k+1}^{a,b}$. This operator plays a key role in understanding persistent structures within the framework of chain complexes. The k*-th persistent Laplacian* is defined as

$$\Delta_k^{a,b} = \partial_{k+1}^{a,b} \circ (\partial_{k+1}^{a,b})^* + (\partial_k^a)^* \circ \partial_k^a. \quad (5)$$

It is important to recognize that the harmonic part of $\Delta_k^{a,b}$, denoted $\ker \Delta_k^{a,b}$, has a natural isomorphism to the (a,b)-persistent homology, expressed as $H_k^{a,b} = \mathrm{im}(H_k(C_*^a) \to H_k(C_*^b))$.[S49] Essentially, the persistent Laplace harmonic component contains information related to persistent homology. To extract valuable information from each chain complex, spectrum analysis is a viable approach. This involves constructing Laplacian matrices for each ∂_k and ∂_{k+1} and analyzing their spectra (eigenvalues and eigenvectors), which can reveal in-depth information about the topological and geometric attributes inherent in the data at a specific filtration scale. The spectral data often offer a concise and informative representation of the data, facilitating effective comparison and analysis across various scales. The persistent attributes used in this work are

shown in Figure S2. This approach underscores the multifaceted nature of persistent topological Laplacians in capturing and representing complex topological and geometric structures.

Element-specific embedding. In this study, the directed flag topological embedding method is used to encode protein-ligand complexes, with the aim of improving the prediction accuracy by better representing the molecular interactions between proteins and ligands. The element-specific topological embedding approach[S28,S50] is utilized to represent these interactions.

When analyzing ligands, attention is focused on heavy elements like carbon (C), nitrogen (N), oxygen (O), sulfur (S), phosphorus (P), fluoride (F), chloride (Cl), bromide (Br), and iodine (I). In the context of proteins, the analysis is limited to carbon (C), nitrogen (N), oxygen (O), and sulfur (S). A series of element combinations, ordered in a specific sequence, are then employed to represent the interactions between the protein and the ligand. For proteins, the combinations are denoted as $\mathcal{E}_{\text{protein}}$={{C}, {N}, {O}, {S}, {C, N}, {C, O}, {C, S}, {N, O}, {N, S}, {O, S}, {C, N, O, S}}. Similarly, the ligand combinations are represented as $\mathcal{E}_{\text{ligand}}$={{C}, {N}, {O}, {S}, {C, N}, {C, O}, {C, S}, {N, O}, {N, S}, {O,S}, {N, P}, {F, Cl, Br, I}, {C, O, N, S, F, P, Cl, Br, I}}. In the Element-specific embedding framework, protein-ligand interactions are delineated through the topological links between two distinct sets of atoms: one set from the protein and the other from the ligand. For instance, a configuration like $K_{\{\text{C,N}\},\{\text{S}\}}$ signifies a directed flag complex where the carbon (C) and nitrogen (N) atoms are part of the protein, while the sulfur (S) atom is part of the ligand. This Element-specific embedding methodology provides a detailed depiction of interactions, focusing on their spatial arrangements and relationships. It can be characterized by distance matrix D as follows,

$$D(i,j) = \begin{cases} \|\mathbf{r}_i - \mathbf{r}_j\|, & \text{if } \mathbf{r_i} \in \mathcal{E}_{\text{protein}}, \mathbf{r_j} \in \mathcal{E}_{\text{ligand}} \text{ or } \mathbf{r_i} \in \mathcal{E}_{\text{ligand}}, \mathbf{r_j} \in \mathcal{E}_{\text{protein}} \\ \infty, & \text{other} \end{cases} \tag{6}$$

where the $\mathbf{r}_i$ and $\mathbf{r}_j$ are coordinates for the ith and jth atoms in the set, and $\|\mathbf{r}_i - \mathbf{r}_j\|$ is their Euclidean distance. In the DFFormer model, the focus is on protein atoms that are situated within

a 20 Å radius of ligand atoms. This model prioritizes protein-ligand interactions by designating an infinite distance value for atom pairs that are exclusively within the protein or within the ligand. This approach ensures that the model's attention is directed specifically towards the interactions between protein and ligand atoms. For any given protein-ligand complex under consideration, there are 143 potential combinations (derived from 11 protein sets multiplied by 13 ligand sets). Each combination represents a unique directed flag complex and is subsequently analyzed using the persistent directed flag Laplacian method. This approach allows for an in-depth examination of the various possible interactions within the protein-ligand complex, leveraging the unique properties of each element set to understand the complex interplay between the protein and ligand at a molecular level.

Topological objects

Graph. The graph is a fundamental structure for illustrating relationships among entities, consisting of nodes (vertices) and edges that connect them. Graphs can be enhanced by adding directionality (directed graphs), assigning weights (weighted graphs), or incorporating geometric properties (geometric graphs). Formally, a graph is defined as a pair (V, E), where V is a set of vertices and E is a subset of $V \times V$ representing edges. Tools such as adjacency matrices, degree matrices, and Laplacian matrices describe interactions between vertices and edges, capturing the graph's topological structure. Although graphs are inherently one-dimensional, simplicial complexes can express their higher-dimensional aspects, including structures such as clique complexes, cell complexes, hypergraphs, neighborhood complexes, and Hom complexes.[S51,S52]

Simplicial complex. A simplicial complex is a mathematical structure that is used to study the shape and topology of the data. It is composed of simplices, which are generalizations of points (0-simplices), line segments (1-simplices), triangles (2-simplices), and their higher-dimensional counterparts. A simplicial complex is formed by combining these simplices in a way that satisfies certain intersection properties: Any face of a simplex in the complex is also a simplex in the

complex, and the intersection of any two simplices is either empty or a shared face. This structure allows for the representation of complex shapes and surfaces by breaking them down into simpler components. Simplicial complexes are widely used in computational topology, particularly in persistent homology, to analyze the multi-scale features of data. They provide a framework for understanding the connectivity, holes, and voids within data, which are crucial for applications in areas such as data analysis, computer graphics, and geometric modeling.

Directed flag complex . The directed flag complex is a combinatorial and topological structure associated with a directed graph (digraph). It generalizes the concept of a simplicial complex to account for the directionality in the underlying graph. Directed flag complexes can be particularly useful in areas like computational topology, where they are used to study data that can be represented as a network or a graph, such as social networks, biological networks, and sensor networks. They provide a way to generalize the concept of a graph to capture not just relationships between individual elements but also more complex relationships involving sequences of elements.

Supplementary Computational Methods and Protocols

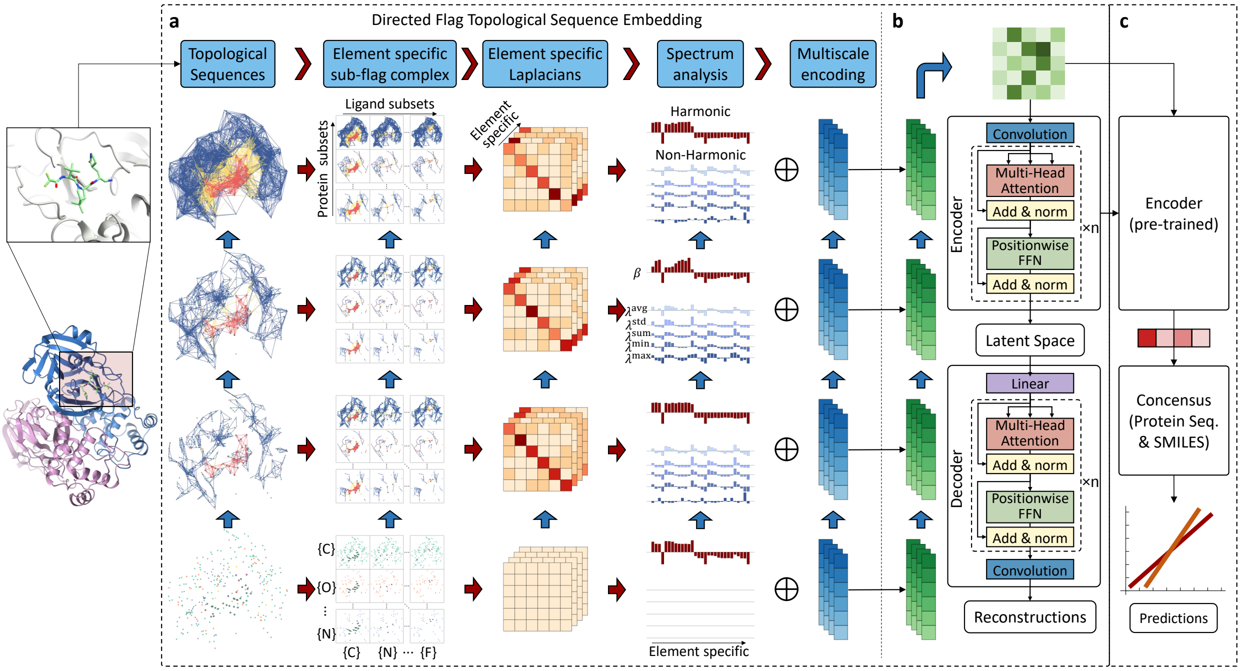

Figure S6: **Schematics of DFFormer.** **a**, A protein-ligand complex is represented by a topological sequence known as a chain complex in algebraic topology. Element-specific sub-complexes are then formed to encode physical interactions at various scales, regulated by a filtration parameter. Element-specific Persistent Topological Directed Flag Laplacians are constructed to extract topological invariants, capturing the shape and stereochemistry of the sub-flag complexes. For these subcomplexes, changes in topological invariants across scales are captured by the harmonic spectrum of the directed flag Laplacians, while their homotopic shape evolution across scales is reflected in the non-harmonic spectrum. Finally, these multiscale topological invariants and homotopic shape (stereochemical) evolutions are compiled into a topological sequence, which serves as input for the transformer. **b**, Self-supervised learning is performed for topological sequences, where the reconstructed sequences are used to compute the loss. **c**, In the supervised fine-tuning stage, the BAs of the protein-ligand complexes are input into the pre-trained encoder. The predictions of DFFormer are combined with those from the sequence-based decision trees to enhance accuracy.

Architecture of the DFFormer model

The DFFormer encoder. DFFormer adopts the architecture of a Masked Autoencoder[S53] (Fig. S1). The Directed Flag Topological Sequence Embedding, once fed into the system, initially passes through a convolution layer. This step is crucial for downsampling the topological embedding and optimizing it for subsequent processing by the encoder module. The convolutional layer is appropriate because the multiscale features from the persistence are related to one another in sequence as the scale parameter varies. Following this convolution layer, the processed embedding advances to multiple encoder layers, each comprising two primary components: a Multi-Head Self-Attention (MHSA) mechanism and a Feed-Forward Neural Network (FFNN). Both components are augmented by normalization layers and incorporate residual connections to enhance the flow of information and learning stability.

The Multi-Head Self-Attention mechanism uses "Scaled Dot-Product Attention". The input of this mechanism consists of queries (Q), keys (K), and values (V), which are all vectors. The attention mechanism is defined as

$$\text{Attention}(Q, K, V) = \text{softmax}\left(\frac{QK^T}{\sqrt{d_k}}\right)V, \tag{7}$$

where d_k is the dimension of the key vectors. This scaling factor $\sqrt{d_k}$ is crucial as it prevents the dot products from growing too large in magnitude, leading to smoother gradients. Specifically, within the framework of DFFormer, each scale's Directed Flag Topological Embedding is adeptly transformed into three distinct vectors: queries (Q), keys (K), and values (V). This transformation is achieved through separate linear transformations, each governed by learnable weights. Subsequently, the attention mechanism is applied to the entire sequence. This process enables each element in the sequence to dynamically attend to every other element, fostering a comprehensive inter-element relationship analysis. Additionally, the Transformer architecture in DFFormer is designed to utilize multiple attention heads, further enhancing its capability to

capture and process complex patterns in the data. Each head learns different aspects of the data:

$$\text{MultiHead}(Q, K, V) = \text{Concat}(\text{head}_1, \text{head}_2, ..., \text{head}_h)W^O, \tag{8}$$

where h is the number of heads, and each head is defined as

$$\text{head}_i = \text{Attention}(QW_i^Q, KW_i^K, VW_i^V) \tag{9}$$

Here, W_i^Q, W_i^K, W_i^V, W^O are parameter matrices.

Beyond the attention mechanism, each encoder layer in the model is equipped with a fully connected feedforward network. This network operates independently and identically on each scale within the layer. This consists of two linear transformations with a GELU (Gaussian Error Linear Unit) activation in between:

$$\text{FFN}(x) = \text{max}(0, xW_1 + b_1)W_2 + b_2 \tag{10}$$

Here, W_1, W_2, b_1, and b_2 are learnable parameters. The GELU activation function introduces non-linearity.

Each sublayer (MHSA and FFNN) in the encoder layer is followed by layer normalization and a residual connection. The output of each sub-layer is

$$\text{Output} = \text{LayerNorm}(x + \text{Sublayer}(x)), \tag{11}$$

where x is the input to the sub-layer, Sublayer(x) is the function implemented by the sub-layer itself (either MHSA and FFNN).

DFFormer decoder. The DFFormer adopts an asymmetric design, which features a more substantial and complex encoder relative to its decoder. The decoder's primary role in the DFFormer is to reconstruct the original directed flag topological sequence embedding from the

latent representations generated by the encoder. This reconstruction process is pivotal because it compels the decoder to accurately regenerate the directed flag topological sequence, thereby driving the encoder to develop rich and insightful representations of the protein-ligand complex data. Such representations are invaluable for a variety of downstream applications.

Structurally, the layers within the DFFormer Decoder closely mirror those of the DFFormer Encoder. The process begins with the encoder's latent representations being linearly mapped. These mappings are then input into the DFFormer Decoders, which are architecturally similar to the encoder layers in the DFFormer Encoder, albeit with a reduced hidden size. During the pretraining phase, the output from the final decoder layer transforms back into the original directed flag topological sequence, facilitated by a convolution layer. In the fine-tuning stage, the focus shifts to the output derived from the first scale's hidden state, which is utilized for regression tasks, such as binding affinity prediction. Specifically, this output is channeled through a linear layer, culminating in generating the final inference output.

Binding affinities and related quantities

This study focuses on the prediction of protein-ligand binding affinities (BAs), which is negative binding free energy ($BA = -\Delta G$). Thus, BA is a positive quantity, and a larger BA indicates stronger binding. The conversion from the inhibition constant K_i in unit of M (mol/l) to BA in unit of kcal/mol is quantified by the equation

$$BA = -RT \ln \frac{K_i}{1} \approx -1.3633 \times \log_{10} \frac{K_i}{1} (kcal/mol), \tag{12}$$

where R is the gas constant, T is the temperature (298 K), 1 denotes the standard concentration 1 M or 1 mol/l, and K_i (in unit of M) can be replaced by IC50 if unavailable.

Sometimes, the binding affinity for protein-ligand complex is expressed as

$$pK_i = -\log_{10} \frac{K_i}{1}, \tag{13}$$

which can be converted to BA via $BA = 1.3633 \times pK_i$. A larger pK_i value indicates stronger protein-ligand binding affinity.

In this work, we also consider the change in binding affinity due to mutation, $\Delta BA = BA_{\text{Mutant}} - BA_{\text{WT}}$, where a negative ΔBA indicates a weakened protein-ligand binding due to the mutation. Similarly, a negative ΔpK_i also indicates weakened binding affinity.

Model evaluation metrics

For model evaluations, we used Pearson Correlation Coefficient (PCC) and root-mean-square errors (RMSEs). The PCC is used to describe the relationship between the variables x and y. It is defined as

$$\mathrm{PCC} = \frac{\sum (x_i - \bar{x})(y_i - \bar{y})}{\sqrt{\sum (x_i - \bar{x})^2 \sum (y_i - \bar{y})^2}}, \tag{14}$$

where x_i represents the value of x in the ith sample, while $\bar{x}$ denotes the average of the x values. Similarly, y_i is the value of y in the ith sample, and $\bar{y}$ is the mean of the y values.

The RMSE is defined as

$$\text{RMSE} = \sqrt{\frac{1}{n}\sum_{i=1}^{n} (y_i - \hat{y}_i)^2}, \tag{15}$$

where y_i and $\hat{y}_i$ are the predicted and true values of ith sample, respectively.

Loss function in model training

The Mean Squared Error (MSE) is used as the loss function throughout the pre-training and fine-tuning stages. MSE calculates the average of the squares of the differences between the predicted and actual values, defined as

$$\text{MSE} = \frac{1}{n}\sum_{i=1}^{n}(y_i - \hat{y}_i)^2, \tag{16}$$

where y_i is the actual value for the i-th data point, $\hat{y}_i$ is the predicted value for the i-th data point, and n is the total number of data points.

Hyperparameter selection and optimization

In the Seq-tree model, the feature embeddings were extracted from large language models ESM for proteins[S54] and the transformer-CPZ model for small molecules[S55] separately. Following this, the Gradient Boosted Decision Trees (GBDT) algorithm from sklearn library was employed for the prediction of protein-ligand binding affinities. The parameters for the GBDT algorithm were set as follows: 'n_estimators' = 10,000, 'max_depth' = 7, 'min_samples_split' = 2, accompanied by a subsample size of 0.4, and a learning rate of 0.005. All other parameters were in the default settings as specified in the algorithm.[S56] During the prediction phase, the LightGBM algorithm was used due to its faster training and better memory efficiency.[S57] The sequence-based features were trained using this algorithm with the following parameters: 'num_boost_round' set = 10000, 'learning_rate' = 0.01, 'num_leaves' = 125, 'feature_fraction' = 0.9, 'bagging_freq' = 5, 'max_depth' = 7, and 'metric' = 'mse'.

In the DFFormer models, a self-supervised learning method was adopted in the pretraining phase, followed by a supervised learning approach in the fine-tuning stage. Similar to the Masked Autoencoder model[S53] in computer vision, DFFormer employs an asymmetric design in its encoder and decoder. The relevant parameters are given in Table S9.

In the pretraining stage, we tested and selected hyperparameters for effective learning and convergence. We used a batch size of 64 and a maximum of 30,000 training steps, along with an initial learning rate of 0.001 to facilitate gradual and effective weight adjustments. To stabilize the early training phase, we incorporated a warm-up period accounting for 5% of the total training steps, where the learning rate gradually increased from 0 to the initial rate. In the subsequent fine-tuning stage, we employed a supervised learning method to refine the model for specific tasks, focusing on preventing overfitting to the hyperparameters. We decreased the batch size to 32 and modified the initial learning rate to 0.0008. The training steps varied for different tasks, setting a maximum of 10,000 steps for the PDBbind 2020 dataset. For the 5-fold cross-validation task on the SARS-CoV/CoV-2 Mpro dataset, we divided the dataset into five folds, training five separate models. Each model was trained using four out of the five folds, building on the model

Table S9: The parameter settings for DFFormer

Parameters	Pretraining stage	Fine-tuning stage
attention_probs_dropout_prob	0.1	0.1
decoder_hidden_size	768	/
decoder_intermediate_size	3072	/
decoder_num_attention_heads	12	/
decoder_num_hidden_layers	8	/
hidden_act	gelu	gelu
hidden_dropout_prob	0.1	0.1
hidden_size	1024	1024
image_size(large)	(100, 143)	(100, 143)
image_size(small)	(50, 143)	(50, 143)
initializer_range	0.02	0.02
intermediate_size	4096	4096
num_attention_heads	4096	4096
num_channels	6	6
num_hidden_layers	12	12
patch_size	(1, 143)	(1, 143)

already fine-tuned with PDBbind 2020. These models underwent 4,000 update steps, with a batch size of 8 and a learning rate of 0.0008. It is important to note that some parameters, such as the warm-up steps and the optimizer, were consistent throughout the pretraining and fine-tuning stages. This consistency supported a cohesive approach to model development. Moreover, during fine-tuning for BA prediction, we consciously limited hyperparameter tuning, which helps avoid overfitting and ensures the effectiveness of mutation scanning.

Supplementary Notes

The PDBbind database is a widely used benchmark for evaluating protein-ligand binding affinities. The core sets from CASF-2007, CASF-2013, and CASF-2016 are commonly employed for validation purposes. However, different studies have used various training sets to develop prediction models. The CASF benchmarks provide the refined set as a standard training dataset to ensure fair comparisons across models. Notably, many previous studies have used the refined set for training and demonstrated impressive accuracy in binding affinity prediction. However, the

performance of these models when trained on larger datasets, such as the general set, remains unclear. This raises questions about their scalability and effectiveness when applied to more diverse protein-ligand complexes.

In this work, the primary goal is to develop a robust predictor for binding affinity, especially for new protein-ligand complexes, such as the Mpro-nirmatrelvir complex and its mutated variants. To achieve this, we utilized a larger training dataset, specifically the general set, to ensure the highest accuracy in binding affinity prediction. Table 1 in the main text highlights recent models trained on the larger general set. For a transparent overview of previous models, Table S10 below presents the binding affinity prediction performance on the PDBbind core sets for models trained on the refined set.

Table S10: Performance (PCC and RMSE) of previous models on the CASF benchmark datasets[a]

Model	CASF-2007	CASF-2013	CASF-2016	PDBbind 2016
Training size	1105	2764	3772	3767
Testing size	195	195	285	290
PerSpect[S58]	0.836 (1.847)	0.793 (1.956)		0.840 (1.724)
TopBP[S50]	0.827 (1.93)	0.808 (1.95)		0.861 (1.65)
AGL-score[S59]	0.83 (1.864)	0.792 (1.973)	0.833 (1.733)	
DC-GBT[S60]	0.824 (1.911)	0.799 (1.952)		0.843 (1.711)
OPRC[S61]	0.821 (1.926)	0.789 (2.010)		0.838 (1.736)
PLEC[S62]		0.757 (2.006)		0.817 (SD=1.712)
Pancy[S63]		0.70 (2.194)		0.78 (1.931)
ChemPLP@GOLD[S64]		0.579 (2.991)		0.614
PMH-Transformer[S65]	0.837 (1.907)	0.807 (1.982)		0.851(1.701)
Δ_{vina}RF[S66]	0.732	0.686		0.816
OnionNet[S67]		0.782 (2.049)		0.816 (1.742)
EISA-score[S68]	0.825 (1.941)	0.756 (2.113)	0.821 (1.835)	
PotentialNet[S69]	0.822 (SD=1.892)			
X-Score::HM[S64]	0.649	0.614 (2.421)		0.609
RF::VinaElem[S70]	0.803 (1.935)			
KDeep[S71]				0.82 (1.731)

[a]The PCC and RMSE (in parentheses, in the unit kcal/mol) for all methods are provided. The refined set of each (year) version of PDBbind[S64,S72–S74] was used as the training set. [b]The CASF-2016 dataset plus five additional protein-ligand complexes (also known as the PDBbind 2016 core set).

References

(S1) Hu, Y.; Lewandowski, E. M.; Tan, H.; Zhang, X.; Morgan, R. T.; Zhang, X.; Jacobs, L. M.; Butler, S. G.; Gongora, M. V.; Choy, J.; others Naturally occurring mutations of SARS-CoV-2 main protease confer drug resistance to nirmatrelvir. *ACS Central Science* **2023**, *9*, 1658–1669.

(S2) Hoffman, R. L.; Kania, R. S.; Brothers, M. A.; Davies, J. F.; Ferre, R. A.; Gajiwala, K. S.; He, M.; Hogan, R. J.; Kozminski, K.; Li, L. Y.; others Discovery of ketone-based covalent inhibitors of coronavirus 3CL proteases for the potential therapeutic treatment of COVID-19. *Journal of medicinal chemistry* **2020**, *63*, 12725–12747.

(S3) Lee, C.-C.; Kuo, C.-J.; Ko, T.-P.; Hsu, M.-F.; Tsui, Y.-C.; Chang, S.-C.; Yang, S.; Chen, S.-J.; Chen, H.-C.; Hsu, M.-C.; others Structural basis of inhibition specificities of 3C and 3C-like proteases by zinc-coordinating and peptidomimetic compounds. *Journal of Biological Chemistry* **2009**, *284*, 7646–7655.

(S4) Dai, W.; Zhang, B.; Jiang, X.-M.; Su, H.; Li, J.; Zhao, Y.; Xie, X.; Jin, Z.; Peng, J.; Liu, F.; others Structure-based design of antiviral drug candidates targeting the SARS-CoV-2 main protease. *Science* **2020**, *368*, 1331–1335.

(S5) Lee, C.-C.; Kuo, C.-J.; Hsu, M.-F.; Liang, P.-H.; Fang, J.-M.; Shie, J.-J.; Wang, A. H.-J. Structural basis of mercury-and zinc-conjugated complexes as SARS-CoV 3C-like protease inhibitors. *FEBS letters* **2007**, *581*, 5454–5458.

(S6) Yang, S.; Chen, S.-J.; Hsu, M.-F.; Wu, J.-D.; Tseng, C.-T. K.; Liu, Y.-F.; Chen, H.-C.; Kuo, C.-W.; Wu, C.-S.; Chang, L.-W.; others Synthesis, crystal structure, structure-activity relationships, and antiviral activity of a potent SARS coronavirus 3CL protease inhibitor. *Journal of medicinal chemistry* **2006**, *49*, 4971–4980.

(S7) Zhang, L.; Lin, D.; Kusov, Y.; Nian, Y.; Ma, Q.; Wang, J.; Von Brunn, A.; Leyssen, P.; Lanko, K.; Neyts, J.; others α-Ketoamides as broad-spectrum inhibitors of coronavirus and

enterovirus replication: structure-based design, synthesis, and activity assessment. *Journal of medicinal chemistry* **2020**, *63*, 4562–4578.

(S8) Lu, I.-L.; Mahindroo, N.; Liang, P.-H.; Peng, Y.-H.; Kuo, C.-J.; Tsai, K.-C.; Hsieh, H.-P.; Chao, Y.-S.; Wu, S.-Y. Structure-based drug design and structural biology study of novel nonpeptide inhibitors of severe acute respiratory syndrome coronavirus main protease. *Journal of medicinal chemistry* **2006**, *49*, 5154–5161.

(S9) Vuong, W.; Khan, M. B.; Fischer, C.; Arutyunova, E.; Lamer, T.; Shields, J.; Saffran, H. A.; McKay, R. T.; van Belkum, M. J.; Joyce, M. A.; others Feline coronavirus drug inhibits the main protease of SARS-CoV-2 and blocks virus replication. *Nature communications* **2020**, *11*, 4282.

(S10) Bacha, U.; Barrila, J.; Gabelli, S. B.; Kiso, Y.; Mario Amzel, L.; Freire, E. Development of broad-spectrum halomethyl ketone inhibitors against coronavirus main protease 3CLpro. *Chemical biology & drug design* **2008**, *72*, 34–49.

(S11) Rathnayake, A. D.; Zheng, J.; Kim, Y.; Perera, K. D.; Mackin, S.; Meyerholz, D. K.; Kashipathy, M. M.; Battaile, K. P.; Lovell, S.; Perlman, S.; others 3C-like protease inhibitors block coronavirus replication in vitro and improve survival in MERS-CoV–infected mice. *Science translational medicine* **2020**, *12*, eabc5332.

(S12) Sacco, M. D.; Ma, C.; Lagarias, P.; Gao, A.; Townsend, J. A.; Meng, X.; Dube, P.; Zhang, X.; Hu, Y.; Kitamura, N.; others Structure and inhibition of the SARS-CoV-2 main protease reveal strategy for developing dual inhibitors against Mpro and cathepsin L. *Science Advances* **2020**, *6*, eabe0751.

(S13) Wang, H.; He, S.; Deng, W.; Zhang, Y.; Li, G.; Sun, J.; Zhao, W.; Guo, Y.; Yin, Z.; Li, D.; others Comprehensive insights into the catalytic mechanism of middle east respiratory syndrome 3C-like protease and severe acute respiratory syndrome 3C-like protease. *ACS catalysis* **2020**, *10*, 5871–5890.

(S14) Zhang, L.; Lin, D.; Sun, X.; Curth, U.; Drosten, C.; Sauerhering, L.; Becker, S.; Rox, K.; Hilgenfeld, R. Crystal structure of SARS-CoV-2 main protease provides a basis for design of improved α-ketoamide inhibitors. *Science* **2020**, *368*, 409–412.

(S15) Su, H.-x.; Yao, S.; Zhao, W.-f.; Li, M.-j.; Liu, J.; Shang, W.-j.; Xie, H.; Ke, C.-q.; Hu, H.-c.; Gao, M.-n.; others Anti-SARS-CoV-2 activities in vitro of Shuanghuanglian preparations and bioactive ingredients. *Acta Pharmacologica Sinica* **2020**, *41*, 1167–1177.

(S16) Verschueren, K. H.; Pumpor, K.; Anemüller, S.; Chen, S.; Mesters, J. R.; Hilgenfeld, R. A structural view of the inactivation of the SARS coronavirus main proteinase by benzotriazole esters. *Chemistry & Biology* **2008**, *15*, 597–606.

(S17) Goetz, D.; Choe, Y.; Hansell, E.; Chen, Y.; McDowell, M.; Jonsson, C.; Roush, W.; McKerrow, J.; Craik, C. Substrate specificity profiling and identification of a new class of inhibitor for the major protease of the SARS coronavirus. *Biochemistry* **2007**, *46*, 8744–8752.

(S18) Zhu, L.; George, S.; Schmidt, M. F.; Al-Gharabli, S. I.; Rademann, J.; Hilgenfeld, R. Peptide aldehyde inhibitors challenge the substrate specificity of the SARS-coronavirus main protease. *Antiviral research* **2011**, *92*, 204–212.

(S19) Turlington, M.; Chun, A.; Tomar, S.; Eggler, A.; Grum-Tokars, V.; Jacobs, J.; Daniels, J. S.; Dawson, E.; Saldanha, A.; Chase, P.; others Discovery of N-(benzo [1, 2, 3] triazol-1-yl)-N-(benzyl) acetamido) phenyl) carboxamides as severe acute respiratory syndrome coronavirus (SARS-CoV) 3CLpro inhibitors: identification of ML300 and noncovalent nanomolar inhibitors with an induced-fit binding. *Bioorganic & medicinal chemistry letters* **2013**, *23*, 6172–6177.

(S20) Yang, H.; Xie, W.; Xue, X.; Yang, K.; Ma, J.; Liang, W.; Zhao, Q.; Zhou, Z.; Pei, D.; Ziebuhr, J.; others Design of wide-spectrum inhibitors targeting coronavirus main proteases. *PLoS biology* **2005**, *3*, e324.

(S21) Chuck, C.-P.; Chen, C.; Ke, Z.; Wan, D. C.-C.; Chow, H.-F.; Wong, K.-B. Design, synthesis and crystallographic analysis of nitrile-based broad-spectrum peptidomimetic inhibitors for coronavirus 3C-like proteases. *European journal of medicinal chemistry* **2013**, *59*, 1–6.

(S22) Shimamoto, Y.; Hattori, Y.; Kobayashi, K.; Teruya, K.; Sanjoh, A.; Nakagawa, A.; Yamashita, E.; Akaji, K. Fused-ring structure of decahydroisoquinolin as a novel scaffold for SARS 3CL protease inhibitors. *Bioorganic & medicinal chemistry* **2015**, *23*, 876–890.

(S23) Ghosh, A. K.; Xi, K.; Ratia, K.; Santarsiero, B. D.; Fu, W.; Harcourt, B. H.; Rota, P. A.; Baker, S. C.; Johnson, M. E.; Mesecar, A. D. Design and synthesis of peptidomimetic severe acute respiratory syndrome chymotrypsin-like protease inhibitors. *Journal of medicinal chemistry* **2005**, *48*, 6767–6771.

(S24) Ghosh, A. K.; Xi, K.; Grum-Tokars, V.; Xu, X.; Ratia, K.; Fu, W.; Houser, K. V.; Baker, S. C.; Johnson, M. E.; Mesecar, A. D. Structure-based design, synthesis, and biological evaluation of peptidomimetic SARS-CoV 3CLpro inhibitors. *Bioorganic & medicinal chemistry letters* **2007**, *17*, 5876–5880.

(S25) Nguyen, D. D.; Gao, K.; Chen, J.; Wang, R.; Wei, G.-W. Unveiling the molecular mechanism of SARS-CoV-2 main protease inhibition from 137 crystal structures using algebraic topology and deep learning. *Chemical science* **2020**, *11*, 12036–12046.

(S26) Clayton, J.; de Oliveira, V. M.; Ibrahim, M. F.; Sun, X.; Mahinthichaichan, P.; Shen, M.; Hilgenfeld, R.; Shen, J. Integrative Approach to Dissect the Drug Resistance Mechanism of the H172Y Mutation of SARS-CoV-2 Main Protease. *Journal of Chemical Information and Modeling* **2023**,

(S27) Iketani, S.; Mohri, H.; Culbertson, B.; Hong, S. J.; Duan, Y.; Luck, M. I.; Annavajhala, M. K.; Guo, Y.; Sheng, Z.; Uhlemann, A.-C.; others Multiple pathways for SARS-CoV-2 resistance to nirmatrelvir. *Nature* **2023**, *613*, 558–564.

(S28) Cang, Z.; Wei, G.-W. TopologyNet: Topology based deep convolutional and multi-task neural networks for biomolecular property predictions. *PLoS computational biology* **2017**, *13*, e1005690.

(S29) Zomorodian, A.; Carlsson, G. Computing persistent homology. Proceedings of the twentieth annual symposium on Computational geometry. 2004; pp 347–356.

(S30) Edelsbrunner; Letscher; Zomorodian Topological persistence and simplification. *Discrete & Computational Geometry* **2002**, *28*, 511–533.

(S31) Wasserman, L. Topological data analysis. *Annual Review of Statistics and Its Application* **2018**, *5*, 501–532.

(S32) Townsend, J.; Micucci, C. P.; Hymel, J. H.; Maroulas, V.; Vogiatzis, K. D. Representation of molecular structures with persistent homology for machine learning applications in chemistry. *Nature communications* **2020**, *11*, 3230.

(S33) Nguyen, D. D.; Cang, Z.; Wu, K.; Wang, M.; Cao, Y.; Wei, G.-W. Mathematical deep learning for pose and binding affinity prediction and ranking in D3R Grand Challenges. *Journal of computer-aided molecular design* **2019**, *33*, 71–82.

(S34) Nguyen, D. D.; Gao, K.; Wang, M.; Wei, G.-W. MathDL: mathematical deep learning for D3R Grand Challenge 4. *Journal of computer-aided molecular design* **2020**, *34*, 131–147.

(S35) Papamarkou, T.; Birdal, T.; Bronstein, M. M.; Carlsson, G. E.; Curry, J.; Gao, Y.; Hajij, M.; Kwitt, R.; Lio, P.; Di Lorenzo, P.; others Position: Topological Deep Learning is the New Frontier for Relational Learning. Forty-first International Conference on Machine Learning. 2024.

(S36) Wei, X.; Wei, G.-W. Persistent Topological Laplacians–a Survey. *arXiv preprint arXiv:2312.07563* **2023**,

(S37) Wang, R.; Nguyen, D. D.; Wei, G.-W. Persistent spectral graph. *International journal for numerical methods in biomedical engineering* **2020**, *36*, e3376.

(S38) Chen, J.; Zhao, R.; Tong, Y.; Wei, G.-W. Evolutionary de rham-hodge method. *Discrete and continuous dynamical systems. Series B* **2021**, *26*, 3785.

(S39) Jones, B.; Wei, G. Persistent Directed Flag Laplacian. *Foundations of Data Science* **doi: 10.3934/fods.2024048, 2024**,

(S40) Wei, X.; Wei, G.-W. Persistent sheaf laplacians. *Foundations of Data Science, doi: 10.3934/fods.2024033* **2024**,

(S41) Wang, R.; Wei, G.-W. Persistent path laplacian. *Foundations of data science (Springfield, Mo.)* **2023**, *5*, 26.

(S42) Chen, D.; Liu, J.; Wu, J.; Wei, G.-W.; Pan, F.; Yau, S.-T. Path topology in molecular and materials sciences. *The Journal of Physical Chemistry Letters* **2023**, *14*, 954–964.

(S43) Liu, X.; Feng, H.; Wu, J.; Xia, K. Persistent spectral hypergraph based machine learning (PSH-ML) for protein-ligand binding affinity prediction. *Briefings in Bioinformatics* **2021**, *22*, bbab127.

(S44) Chen, D.; Liu, J.; Wu, J.; Wei, G.-W. Persistent hyperdigraph homology and persistent hyperdigraph Laplacians. *Foundations of Data Science* **2023**, *5*, 558–588.

(S45) Horak, D.; Jost, J. Spectra of combinatorial Laplace operators on simplicial complexes. *Advances in Mathematics* **2013**, *244*, 303–336.

(S46) Eckmann, B. Harmonische funktionen und randwertaufgaben in einem komplex. *Commentarii Mathematici Helvetici* **1944**, *17*, 240–255.

(S47) Chen, J.; Zhao, R.; Tong, Y.; Wei, G.-W. Evolutionary de Rham-Hodge method. *arXiv preprint arXiv:1912.12388* **2019**,

(S48) Mémoli, F.; Wan, Z.; Wang, Y. Persistent Laplacians: Properties, algorithms and implications. *SIAM Journal on Mathematics of Data Science* **2022**, *4*, 858–884.

(S49) Liu, J.; Li, J.; Wu, J. The algebraic stability for persistent Laplacians. *arXiv preprint arXiv:2302.03902* **2023**,

(S50) Cang, Z.; Mu, L.; Wei, G.-W. Representability of algebraic topology for biomolecules in machine learning based scoring and virtual screening. *PLoS computational biology* **2018**, *14*, e1005929.

(S51) Kozlov, D. *Combinatorial algebraic topology*; Springer Science & Business Media, 2008; Vol. 21.

(S52) Lovász, L. Kneser's conjecture, chromatic number, and homotopy. *Journal of Combinatorial Theory, Series A* **1978**, *25*, 319–324.

(S53) He, K.; Chen, X.; Xie, S.; Li, Y.; Dollár, P.; Girshick, R. Masked autoencoders are scalable vision learners. Proceedings of the IEEE/CVF conference on computer vision and pattern recognition. 2022; pp 16000–16009.

(S54) Rives, A.; Meier, J.; Sercu, T.; Goyal, S.; Lin, Z.; Liu, J.; Guo, D.; Ott, M.; Zitnick, C. L.; Ma, J.; others Biological structure and function emerge from scaling unsupervised learning to 250 million protein sequences. *Proceedings of the National Academy of Sciences* **2021**, *118*, e2016239118.

(S55) Chen, D.; Zheng, J.; Wei, G.-W.; Pan, F. Extracting predictive representations from hundreds of millions of molecules. *The journal of physical chemistry letters* **2021**, *12*, 10793–10801.

(S56) Pedregosa, F.; Varoquaux, G.; Gramfort, A.; Michel, V.; Thirion, B.; Grisel, O.; Blondel, M.; Prettenhofer, P.; Weiss, R.; Dubourg, V.; others Scikit-learn: Machine learning in Python. *the Journal of machine Learning research* **2011**, *12*, 2825–2830.

(S57) Ke, G.; Meng, Q.; Finley, T.; Wang, T.; Chen, W.; Ma, W.; Ye, Q.; Liu, T.-Y. Lightgbm: A highly efficient gradient boosting decision tree. *Advances in neural information processing systems* **2017**, *30*.

(S58) Meng, Z.; Xia, K. Persistent spectral–based machine learning (PerSpect ML) for protein-ligand binding affinity prediction. *Science advances* **2021**, *7*, eabc5329.

(S59) Nguyen, D. D.; Wei, G.-W. AGL-score: algebraic graph learning score for protein–ligand binding scoring, ranking, docking, and screening. *Journal of chemical information and modeling* **2019**, *59*, 3291–3304.

(S60) Liu, X.; Feng, H.; Wu, J.; Xia, K. Dowker complex based machine learning (DCML) models for protein-ligand binding affinity prediction. *PLoS computational biology* **2022**, *18*, e1009943.

(S61) Wee, J.; Xia, K. Ollivier persistent Ricci curvature-based machine learning for the protein–ligand binding affinity prediction. *Journal of Chemical Information and Modeling* **2021**, *61*, 1617–1626.

(S62) Wójcikowski, M.; Kukiełka, M.; Stepniewska-Dziubinska, M. M.; Siedlecki, P. Development of a protein–ligand extended connectivity (PLEC) fingerprint and its application for binding affinity predictions. *Bioinformatics* **2019**, *35*, 1334–1341.

(S63) Stepniewska-Dziubinska, M. M.; Zielenkiewicz, P.; Siedlecki, P. Development and evaluation of a deep learning model for protein–ligand binding affinity prediction. *Bioinformatics* **2018**, *34*, 3666–3674.

(S64) Su, M.; Yang, Q.; Du, Y.; Feng, G.; Liu, Z.; Li, Y.; Wang, R. Comparative assessment of scoring functions: the CASF-2016 update. *Journal of chemical information and modeling* **2018**, *59*, 895–913.

(S65) Feng, H.; Shen, L.; Liu, J.; Wei, G.-W. Mayer-homology learning prediction of protein-ligand binding affinities. *arXiv preprint arXiv:2408.13299* **2024**,

(S66) Wang, C.; Zhang, Y. Improving scoring-docking-screening powers of protein–ligand scoring functions using random forest. *Journal of computational chemistry* **2017**, *38*, 169–177.

(S67) Zheng, L.; Fan, J.; Mu, Y. Onionnet: a multiple-layer intermolecular-contact-based convolutional neural network for protein–ligand binding affinity prediction. *ACS omega* **2019**, *4*, 15956–15965.

(S68) Rana, M. M.; Nguyen, D. D. EISA-Score: Element Interactive Surface Area score for protein–ligand binding affinity prediction. *Journal of Chemical Information and Modeling* **2022**, *62*, 4329–4341.

(S69) Feinberg, E. N.; Sur, D.; Wu, Z.; Husic, B. E.; Mai, H.; Li, Y.; Sun, S.; Yang, J.; Ramsundar, B.; Pande, V. S. PotentialNet for molecular property prediction. *ACS central science* **2018**, *4*, 1520–1530.

(S70) Li, H.; Leung, K.-S.; Wong, M.-H.; Ballester, P. J. Improving AutoDock Vina using random forest: the growing accuracy of binding affinity prediction by the effective exploitation of larger data sets. *Molecular informatics* **2015**, *34*, 115–126.

(S71) Jiménez, J.; Skalic, M.; Martinez-Rosell, G.; De Fabritiis, G. K deep: protein–ligand absolute binding affinity prediction via 3d-convolutional neural networks. *Journal of chemical information and modeling* **2018**, *58*, 287–296.

(S72) Liu, Z.; Li, Y.; Han, L.; Li, J.; Liu, J.; Zhao, Z.; Nie, W.; Liu, Y.; Wang, R. PDB-wide collection of binding data: current status of the PDBbind database. *Bioinformatics* **2015**, *31*, 405–412.

(S73) Cheng, T.; Li, X.; Li, Y.; Liu, Z.; Wang, R. Comparative assessment of scoring functions on a diverse test set. *Journal of chemical information and modeling* **2009**, *49*, 1079–1093.

(S74) Li, Y.; Han, L.; Liu, Z.; Wang, R. Comparative assessment of scoring functions on an updated benchmark: 2. Evaluation methods and general results. *Journal of chemical information and modeling* **2014**, *54*, 1717–1736.
